# Characterization of initial ankle-foot prosthesis prescription patterns in U.S. Service members following unilateral transtibial amputation

**DOI:** 10.3389/fresc.2023.1235693

**Published:** 2023-08-25

**Authors:** Patrick G. Monaghan, Ashley D. Knight, Sarah A. Brinkerhoff, Kenneth D. Harrison, Christopher L. Dearth, Brad D. Hendershot, JoEllen M. Sefton, Michael Zabala, Adan Vazquez, David Shannon, David Crumbley, Jaimie A. Roper

**Affiliations:** ^1^ School of Kinesiology, Auburn University, Auburn, AL, United States; ^2^ Research & Surveillance Section, Extremity Trauma and Amputation Center of Excellence, Defense Health Agency, Falls Church, VA, United States; ^3^ Department of Rehabilitation, Walter Reed National Military Medical Center, Bethesda, MD, United States; ^4^ Department of Physical Medicine & Rehabilitation, Uniformed Services University of the Health Sciences, Bethesda, MD, USA; ^5^ Department of Surgery, Uniformed Services University of the Health Sciences—Walter Reed National Military Medical Center, Bethesda, MD, United States; ^6^ Department of Mechanical Engineering, Auburn University, Auburn, AL, United States; ^7^ Department of Prosthetics and Orthotics, Alabama State University, Montgomery, AL, United States; ^8^ Department of Educational Foundations, Leadership, and Technology, Auburn University, Auburn, AL, United States; ^9^ School of Nursing, Auburn University, Auburn, AL, United States

**Keywords:** military, lower-limb, device, prosthetic, rehabilitation

## Abstract

**Introduction:**

The purpose of this study was to explore relationships between patient-specific characteristics and initial ankle-foot prosthesis prescription patterns among U.S. Service members with unilateral transtibial limb loss.

**Methods:**

A retrospective review of health records identified 174 individuals with unilateral transtibial limb loss who received care at Walter Reed National Military Medical Center between 2001 and 2019. We examined patient-specific factors such as demographics, participant duty status at injury and amputation, amputation etiology, and timing between injury, amputation, and initial prescription. The type of first prescribed ankle-foot prosthesis was categorized as energy storing and return - nonarticulating, energy storing and return - articulating, or computer controlled.

**Results:**

Sex, amputation etiology, time from injury to initial prescription, and time from amputation to initial prescription differed by type of initial ankle-foot prosthesis prescription. Service members with shorter intervals between injury-initial prescription and amputation-initial prescription, and those injured by combat blast, were more likely to receive a non-articulating device. Incorporating sex, time from injury-initial prescription, time from amputation-initial prescription, and amputation etiology as predictors of prosthesis type, we were able to correctly classify 72% of all first prostheses prescribed.

**Discussion:**

Patient-specific characteristics such as sex, the time between injury-initial prescription, time from amputation-initial prescription and amputation etiology are essential characteristics that influence initial ankle-foot prosthesis prescription patterns in U.S. Service members.

## 1. Introduction

Projections estimate that by 2050, the number of persons with limb loss in the United States (U.S.) will more than double to 3.6 million (1), largely due to dysvascular disease. Between 2001 and 2017, 1705 U.S. Service members sustained 1914 total deployment-related (major) amputations (2). Lower limb amputations account for 86% of all amputations within the United States(3); specifically, transtibial amputations are the most common form of lower limb loss, and account for 52% of all amputations in U.S. Service members (2, 4). Use of a lower limb prosthesis improves the quality of life and mobility for individuals with lower limb loss (5). Yet, while prosthetic device prescription is critical in achieving optimal outcomes (6), there remains minimal evidence to guide optimal device selection. Ankle-foot prosthesis prescription is a challenging and complex process, compounded with the abundance of commercially available ankle-foot componentry (7). Previous studies have highlighted insufficient evidence from high-quality comparative studies to develop or establish criteria for the prescription of prosthetic ankle-foot devices (8). Consequently, prescription tends to be primarily governed by the professional judgment of the limb loss care team (4).Patient-specific factors likely play a role in prosthesis prescription. For example, recently the US Department of Veterans Affairs and the US Department of Defense developed clinical practice guidelines for rehabilitation of lower limb amputation to address key clinical questions. One of the key outcomes was the need to consider what factors (demographic, clinical, biologic, environment, socioeconomic) are associated with better outcomes (9). However, individuals with lower limb loss exhibit marked heterogeneity regarding specific demographics and possess distinct medical and injury histories. For example, 72% of transtibial amputations among civilians are attributable to dysvascular etiologies, while only 7% are trauma-related (3, 4). Whereas, trauma-related injuries are the most prevalent cause of limb loss among U.S. Service members (2, 10); 90% of the 1914 major limb amputations reported between 2001 and 2017 amongst U.S. Service members were attributed to traumatic blast injury (2). Moreover, the particular type of trauma experienced, such as a gunshot wound, motor vehicle accident/crash, or an explosive blast, can influence the surgical procedure and prosthetic prescription process (11). The mechanistic underpinnings of the injury are important to consider in device prescription and may improve outcomes. The interval between the occurrences of injury, the resultant amputation procedure, and the initial prosthetic fitting directly influences outcomes. Although many amputations in combat-related settings occur acutely (within 3 months of injury), Stinner and colleagues report that out of 348 major limb amputations, 15% of those procedures occurred three months post-injury (12). Delayed amputations (amputations occurring greater than 48 h after admission) can increase disability and lead to poorer psychological and functional outcomes (13, 14). For example, Melcer and colleagues highlighted that patients treated with late as opposed to early amputations following combat injuries demonstrated higher rates of adverse physical and psychological outcomes (15). Further, patients who receive an amputation three months post-injury experience reduced functional outcomes at two years compared to groups who receive the amputation closer to the time of injury (16). The time between amputation and initial prosthetic prescription may also influence patient satisfaction with their prosthetic device and the frequency of use. Previous reports have documented that 43% of Veterans with transtibial amputation were fit with a prosthesis within 10.3 months of amputation, with this prescription rate increasing to 52% within 17.5 months (17). Receipt of the first prosthetic device greater than 60-days post-amputation is strongly related to less frequent device use and less satisfaction regarding the prosthesis fit, comfort, appearance, and overall performance (18). Early prosthetic prescription has many physical and psychological benefits (19). Many commercial ankle-foot componentry devices are available for the limb loss care team to prescribe (7), including articulating and non-articulating energy-storage-and-return (ESR) devices. Despite the lack of clear clinical guidelines, ESR is among the most commonly prescribed ankle-foot devices within the Department of Defense (DoD) (20). Using ESR devices can offer several advantages, such as increasing walking speed, reducing the energetic cost of walking, improving elastic response, and aiding propulsion (21–23). Various non-articulating ESR ankle-foot devices can have different mechanical characteristics; however, studies have demonstrated no significant differences in functional outcomes between these non-articulating ESR ankle-foot devices (24). Furthermore, the use of a microprocessor-controlled ankle-foot device has demonstrated improved ambulation during stair ascent (25). While studies have compared articulating ankle-foot devices with other categories of ankle-foot devices, few have reported significant differences regarding functional outcomes. While individual goals and the functional status of the person are crucial to consider within the prescription process, there remains insufficient evidence to support the prescription of specific prosthetic ankle-foot devices within these overarching classifications (26). The purpose of this study was to retrospectively describe the types and sequence/timing of the initial prescribed ankle-foot prosthesis(es), and evaluate corresponding relationships with patient-specific demographics and injury characteristics in U.S. Service members receiving care within the Department of Defense at Walter Reed National Military Medical Center. Defining such relationships will provide a necessary first step toward developing patient-specific strategies and provide an evidence-based benchmark for selecting an ankle-foot prosthesis.. Failure to consider these factors may reduce treatment effectiveness, amplify disability, and decrease device satisfaction/use.

## 2. Materials and methods

### 2.1. Experimental design and procedures

This study consisted of a retrospective analysis of electronic health records of U.S. Service members with unilateral transtibial limb loss who received care at Walter Reed National Military Medical Center between January 1, 2001 and September 1, 2019. Research personnel reviewed the electronic health records to identify and extract participant demographics and relevant medical history, as well as all prosthetic devices received. A total of 305 U.S. Service members and/or dependents with unilateral transtibial limb loss were identified; of these, 131 were removed due to missing data (e.g., injury/amputation timing, could not adequately characterize prosthetic devices received), resulting in a final sample of 174 participants (Supplementary Table S5). This study was approved by the Institutional Review Boards at Walter Reed National Military Medical Center and Auburn University.

### 2.2. Outcome measures

Participant demographics are provided in Table 1 and include age at the injury that resulted in amputation, age at ankle-foot prosthesis prescription, and sex. Participants' duty status at the time of injury and amputation were also collected and classified as active, retired, or active reserve, as well as dependent. The amputation etiology was also recorded and classified as combat blast injury, non-combat blast injury, motor vehicle accident, dysvascular causes, cancer, or other. Details related to prosthetic devices were extracted, including the make, model, and corresponding delivery timeline/sequence (i.e., to define first and/or subsequent devices). Instructions for use and specification documents were derived from the manufacturers' manual (see supplemental material). Prosthetic devices were categorized into groups based on type, function, and features into three overarching groups; (1) energy-storage-and-return and non-articulating (ESR-NA; passive, flexible/dynamic elastic response), (2) energy-storage-and-return storing and articulating (ESR-AR; passive, mechanical articulation, hinged ankle), and (3) computer-controlled (COMP; active/adaptive articulation with use of software, sensors, batteries, etc.). Note, the COMP category includes ankle-foot devices with powered propulsion. Prosthetic ankle-foot devices included in this investigation were classified at the time of study initiation by a diverse panel of experts including prosthetists and limb loss researchers. The panel considered device structure, componentry, function, and biomechanical properties to create ankle-foot device categories. More specific characteristics and features of the initial ankle-foot devices, including the subtype make and model, can be observed in Supplementary Table S4 (Supplementary Material). Ankle-foot device prescriptions were formalized by a physician, considering input from a variety of multidisciplinary team members. The number of days between i) injury and amputation, ii) injury to initial prosthesis prescription, and iii) amputation to initial prosthesis prescription were also calculated. For persons with amputations secondary to vascular disease and cancer, the time from injury to amputation was calculated as the number of days between the reported date of diagnosis of the condition and the reported date of amputation. It is also crucial to highlight that the date of diagnosis may not correspond to the actual onset of the disease process, as there may be a significant time between the condition onset and diagnosis date. However, due to the nature of our study design, the date of diagnosis was the most appropriate and accurate index to include to capture this outcome.

**Table 1:** Sample demographics, by device type of first prescribed ankle-foot prosthesis.

		All duty categories	Active duty	Not active duty
All foot categories	*N*	*174*	*155*	*19*
	Age at injury	29.1 (11.6)	26.1 (5.9)	53.5 (16.8)
	Age at prescription	31.3 (11.7)	28.3 (6.6)	55.1 (16.7)
	Days from amputation to prescription	311.7 (689.3)	330.5 (726.5)	158.3 (153.1)
	Days from injury to amputation	307.0 (661.7)	327.2 (693.8)	142.6 (240.0)
	Days from injury to prescription	631.3 (921.1)	657.7 (953.7)	416.3 (563.5)
	Sex	163 Male	151 Male	12 Male
		11 Female	4 Female	7 Female
	Duty status at injury	155 Active Duty	150 Active Duty	1 Active Reserve
		1 Active Reserve		6 Dependent
		6 Dependent		12 Retired
		12 Retired		
	Amputation etiology	6 Cancer	1 Cancer	5 Cancer
		109 Combat blast	109 Combat blast	10 Dys. disease
		10 Dys. disease	15 Gunshot	1 Gunshot
		16 Gunshot	16 Motor vehicle	1 Motor vehicle
		17 Motor vehicle	14 Other	2 Other
		16 Other		
ESR-NA	*N*	*138*	*127*	*11*
	Age at injury	28.0 (10.9)	25.7 (5.6)	54.3 (20.1)
	Age at prescription	30.1 (11.1)	27.8 (6.2)	55.8 (19.8)
	Days from amputation to prescription	271.4 (624.4)	280.5 (648.1)	166.6 (190.4)
	Days from injury to amputation	273.0 (585.2)	288.9 (605.8)	89.8 (164.4)
	Days from injury to prescription	560.3 (841.9)	569.4 (856.1)	455.7 (679.9)
	Sex	129 Male	124 Male	5 Male
		6 Female	3 Female	3 Female
	Duty status at injury	127 Active Duty	124 Active Duty	1 Active Reserve
		1 Active Reserve		3 Dependent
		3 Dependent		7 Retired
		7 Retired		
	Amputation etiology	2 Cancer	92 Combat blast	2 Cancer
		92 Combat blast	12 Gunshot	6 Dys. disease
		6 Dys. disease	13 Motor vehicle	1 Gunshot
		13 Gunshot	10 Other	2 Other
		13 Motor vehicle		
		12 Other		
ESR-AR	*N*	*28*	*21*	*7*
	Age at injury	33.5 (14.5)	26.7 (6.8)	53.9 (12.2)
	Age at prescription	36.8 (14.1)	30.7 (8.0)	55.2 (12.6)
	Days from amputation to prescription	464.9 (988.2	579.8 (1,123.4)	120.1 (54.3)
	Days from injury to amputation	533.2 (988.2)	666.4 (1,108.0)	133.7 (231.5)
	Days from injury to prescription	998.1 (1,269.7)	1,246.2 (1,377.6)	253.9 (257.1)
	Sex	25 Male	21 Male	4 Male
		3 Female		3 Female
	Duty status at injury	21 Active Duty	21 Active duty	3 Dependent
		3 Dependent		4 Retired
		4 Retired		
	Amputation etiology	3 Cancer	13 Combat blast	3 Cancer
		13 Combat blast	3 Gunshot	1 Dys. disease
		4 Dys. disease	1 Motor vehicle	
		3 Gunshot	4 Other	
		1 Motor vehicle		
		4 Other		
COMP	*N*	*8*	*7*	*1*
	Age at injury	31.5 (7.6)	30.0 (6.8)	42
	Age at prescription	33.4 (8.1)	31.7 (6.9)	45.7
	Days from amputation to prescription	469.6 (437.3)	489 (468.6)	334
	Days from injury to amputation	102.0 (276.5)	4.3 (7.4)	786
	Days from injury to prescription	571.6 (484.8)	493.3 (465.7)	1,120.00
	Sex	6 Male	6 Male	1 Female
		2 Female	1 Female	
	Duty status at injury	7 Active Duty	7 Active Duty	1 Retired
		1 Retired		
	Amputation etiology	1 Cancer	1 Cancer	1 Motor vehicle
		4 Combat blast	4 Combat blast	
		3 Motor vehicle	2 Motor vehicle	

Values for continuous outcomes are given in Mean (Standard Deviation) [minimum, maximum]. Values for categorical outcomes are given in frequency counts. ESR-NA, energy-storage-and-return and non-articulating; ESR-AR, energy-storage-and-return and articulating; COMP, computer-controlled.

### 2.3. Statistical analyses

Age at injury, age at prescription, time from injury to amputation, time from injury to first prescription, and time from amputation to first prescription were assessed for normality using a Shapiro-Wilk test. The distributions of all five outcome measures were skewed right and departed from normality (Age at injury, W = 0.729, *p* < 0.001; age at prescription, W = 0.777, *p* < 0.001; time from injury to amputation, W = 0.533, *p* < 0.001; time from injury to first prescription, W = 0673, *p* < 0.001; time from amputation to first prescription, W = 0.425, *p* < 0.001). Therefore, age at injury, age at prescription, time from injury to amputation, time from injury to first prescription, and time from amputation to first prescription were categorized by the median based on their distributions and compared to the type of first device with the Pearson's Chi-square test. Sex, military status at injury, and amputation etiology were also compared to the type of first device with the Pearson's chi-square test. We then estimated the probability of the first prescribed ankle-foot prosthesis type using a multinomial logistic regression model, where ESR-NA was the reference category. Of the 174 participants, 138 were first prescribed an ESR-NA, 28 an ESR-AR, and 8 a COMP. The COMP category included 7 passive and 1 powered ankle-foot device. The sample size for those prescribed an ESR-NA was considerably larger than for the other two device types; therefore, we used choice-based sampling (27) to randomly sample 28 patients of the 138 prescribed an ESR-NA to represent the sample in the multinomial logistic regression model. Therefore, the multinomial logistic regression model included 64 patients: 28 prescribed an ESR-NA, 28 prescribed an ESR-AR, and 8 prescribed a COMP. No differences were observed between the choice-based sample of *n* = 28 for ESR-NA and the original sample of *n* = 138 (comparison of demographic information can be seen in Supplementary Table S6). Regression model predictors included any measures that significantly differed by type of first prescribed device according to the Pearson's Chi-square tests above. A *p*-value of less than 0.05 was considered to indicate statistical significance.

## 3. Results

**Figure 1:**
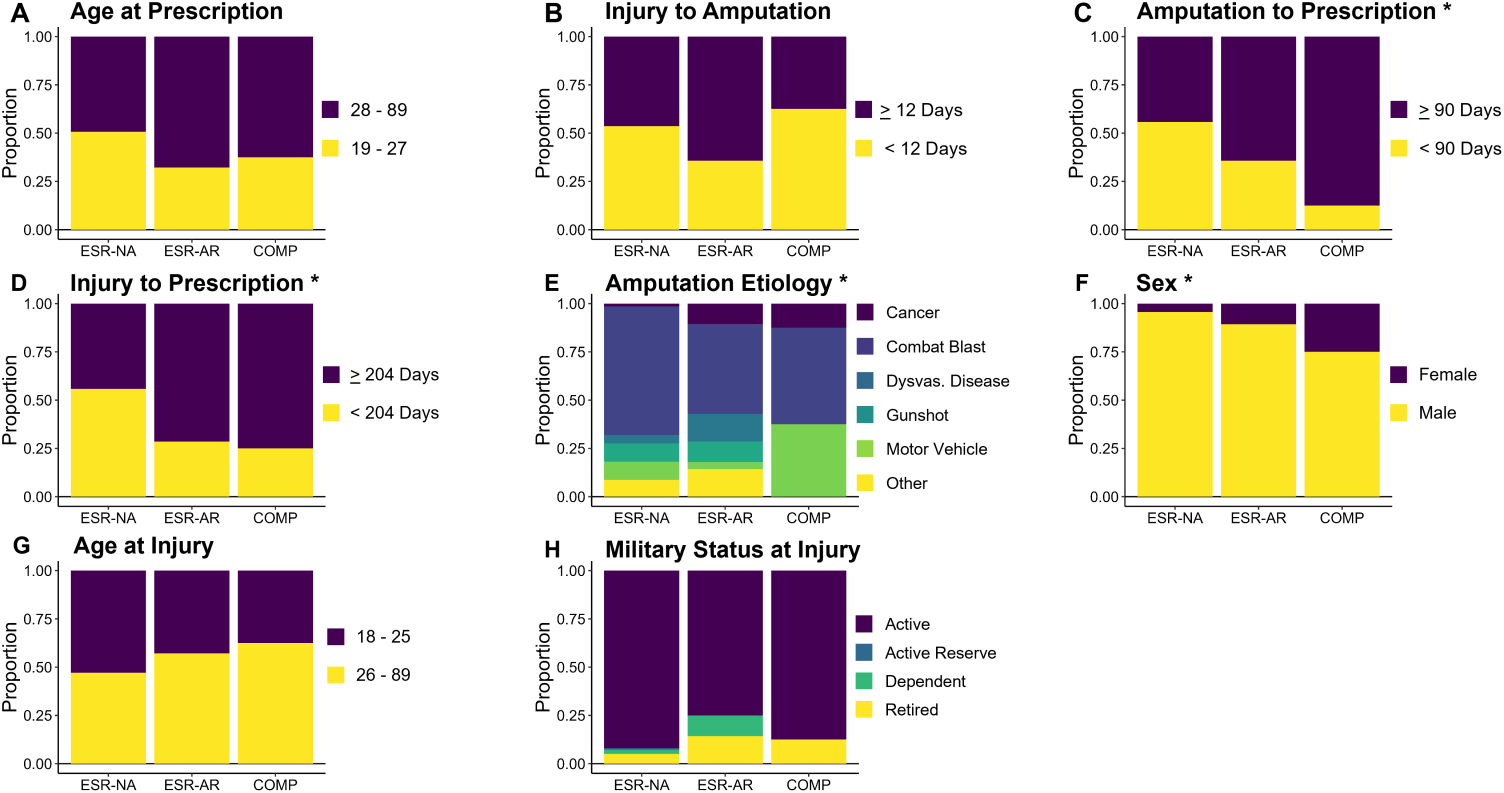
Proportion of patients prescribed an ESR-NA, ESR-AR, and COMP as their first device by (**A**) age at prescription, (**B**) time from injury to amputation, (**C**) time from injury to prescription, (**D**) time from amputation to prescription, (**E**) amputation etiology, (**F**) sex, (**G**) age at Injury and (**H**) military status at injury. *N* = 174; *n* = 138 ESR-NA; *n* = 28 ESR-AR; *n* = 8 COMP. ESR-NA, energy-storage-and-return and non-articulating; ESR-AR, energy-storage-and-return and articulating; COMP, computer-controlled. Asterisks indicate significant chi-squared tests; *p < 0.05, **p < 0.01.

**Figure 2:**
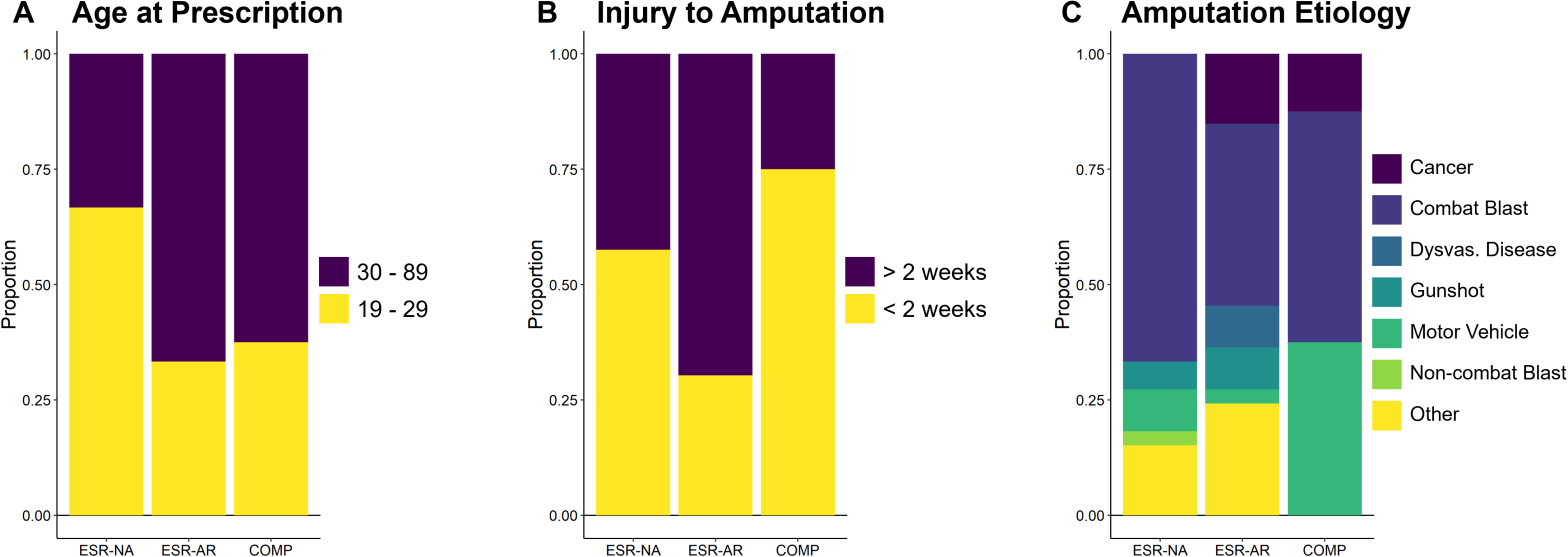
Proportion of patients prescribed an ESR-NA, ESR-AR, and COMP as their first device by (**A**) age at prescription, (**B**) time from injury to amputation, and (**C**) MOI. *N* = 64; *n* = 28 ESR-NA; *n* = 28 ESR-AR; *n* = 8 COMP. ESR-NA, energy-storage-and-return and non-articulating; ESR-AR, energy-storage-and-return and articulating; COMP, computer-controlled.

In the full sample of 174 patients, 4 of the 8 measures differed by the first device prescribed. Time from injury to initial prescription [*Χ*^2^(2) = 8.998, *p* = 0.011] and time from amputation to initial prescription [*Χ*^2^(2) = 8.619, *p* = 0.013] differed such that those prescribed an ESR-AR or a COMP were more likely to be prescribed their device later than those prescribed an ESR-NA (Figure 1). Amputation etiology differed such that those prescribed a COMP were most likely to have been injured by a motor vehicle, while those prescribed an ESR-NA were most likely to have been injured by a combat blast and least likely to have cancer as the cause of amputation [*Χ*^2^(12) = 24.294, *p* = 0.019]. Finally, people prescribed COMP devices were more likely to be female than people prescribed an ESR-AR or an ESR-NA [*Χ*^2^(2) = 6.533, *p* = 0.038]. The proportion of each device by these variables can be seen in Figure 2. Age at injury [*Χ*^2^(2) = 1.512, *p* = 0.469], age at prescription [*Χ*^2^(2) = 3.537, *p* = 0.171], time from injury to amputation [*Χ*^2^(2) = 3.420, *p* = 0.181], and participant duty status at injury [*Χ*^2^(2) = 9.481, *p* = 0.148] did not differ by type of first device prescribed. Therefore, the multinomial logistic regression model to predict the type of first device prescribed was stratified by time from injury to initial prescription, time from amputation to initial prescription, sex, and amputation etiology. The model correctly classified the first device prescribed for 46 out of 64 patients (72%) and misclassified 18 patients (28%). The model correctly classified 71% of patients prescribed an ESR-NA; 71% of patients prescribed an ESR-AR, and 38% of patients prescribed a COMP (Table 2). The odds that a patient would be prescribed an ESR-AR over an ESR-NA were affected by amputation etiology, such that those injured by a motor vehicle accident or a combat blast were less likely to be prescribed an ESR-AR than an ESR-NA, and those amputated as a result of dysvascular disease, cancer, gunshot, or other mechanism were more likely to be prescribed an ESR-AR than an ESR-NA. The probabilities and *p*-values that a patient would be prescribed an ESR-AR over an ESR-NA are shown in Table 3. The odds that a patient would be prescribed a COMP over an ESR-NA were affected by amputation etiology and sex, such that those who were injured by dysvascular disease, gunshot, or other mechanism were less likely to be prescribed a COMP than an ESR-NA, and those injured by cancer, combat blast, or motor vehicle accident were more likely to be prescribed a COMP than an ESR-NA. Sex affected the odds such that females were more likely to be receive a COMP than an ESR-NA. The probabilities and *p*-values that a patient would be prescribed a COMP vs. an ESR-NA are shown in Table 3.

**Table 2:** Predicted versus actual classifications by device type of first prescribed ankle-foot prosthesis.

		Actual			
		ESR-NA	ESR-AR	COMP	
Predicted	ESR-NA	20 (71%)	8	3	31
	ESR-AR	8	20 (71%)	2	30
	COMP	0	0	3 (38%)	3
		28	28	8	

The shaded cells are those devices correctly predicted by the multinomial logistic regression model. ESR-NA, energy-storage-and-return and non-articulating; ESR-AR, energy-storage-and-return and articulating; COMP, computer-controlled.

**Table 3:** The probability that a patient would be prescribed an ESR-AR or a COMP versus an ESR-NA device.

Probability of being prescribed an ESR-AR over an ESR-NA		
Outcome	Probability	*p*-value
Days from injury to prescription = less than 204 days	0.26	0.151
Days from injury to prescription = at least 204 days	0.74	
Days from injury to amputation = less than 12 days	0.30	0.196
Days from injury to amputation = at least 12 days	0.70	
Amputation etiology = cancer	>0.99	**<0.001**
Amputation etiology = combat blast	<0.01	**<0.001**
Amputation etiology = dysvascular disease	0.85	**<0.001**
Amputation etiology = gunshot	0.58	**<0.001**
Amputation etiology = motor vehicle accident	0.31	**<0.001**
Amputation etiology = other	0.65	**<0.001**
Sex = Male	0.84	0.324
Sex = Female	0.16	
Probability of being prescribed a COMP over an ESR-NA		
Outcome	Probability	*p*-value
Days from injury to prescription = less than 204 days	0.25	0.286
Days from injury to amputation = at least 204 days	0.75	
Days from injury to amputation = less than 12 days	0.62	0.628
Days from injury to amputation = at least 12 days	0.38	
Amputation etiology = cancer	>0.99	**<0.001**
Amputation etiology = combat blast	>0.99	**<0.001**
Amputation etiology = dysvascular disease	<0.01	**<0.001**
Amputation etiology = gunshot	<0.01	**<0.001**
Amputation etiology = motor vehicle accident	0.74	**<0.001**
Amputation etiology = other	<0.01	**<0.001**
Sex = Male	<0.01	**<0.001**
Sex = Female	>0.99	

Values in bold indicate significant probabilities. ESR-NA, energy-storage-and-return and non-articulating; ESR-AR, energy-storage-and-return and articulating; COMP, computer-controlled.

## 4. Discussion

This study aimed to characterize initial ankle-foot prosthesis prescription patterns within U.S. Service members and dependents with unilateral transtibial limb loss receiving care within the Department of Defense at Walter Reed National Military Medical Center. It is important to note that we analyzed initial device prescription, which may not be representative of the optimal outcome, yet still an important distinction within the prescription process. We first explored this relationship in the entire sample (*N* = 174) and then examined it with a more even distribution amongst ankle-foot prosthesis types in our final model (*N* = 68). We also assessed the ability of our final model to predict and correctly classify the initial ankle-foot prosthesis prescribed. We report three main findings from the final model: (1) initial ankle-foot prosthesis prescription differed by time from injury to initial prescription, time from amputation to initial prescription, sex, and amputation etiology; (2) incorporating time from injury to initial prescription, time from amputation to initial prescription, sex, and amputation etiology as predictors of initial ankle-foot prosthesis type, we were able to correctly classify 72% of all first prostheses prescribed; (3) females were more likely to have first prescriptions of COMP devices. The time between injury and initial prescription, and the time between amputation and initial prescription impacted the choice of the first ankle-foot prosthesis. The mean (SD) number of days between injury and initial ankle-foot prescription for those receiving an ESR-NA prosthesis was 560 (842) days, 998 (1,270) days for ESR-AR prosthesis, and 572 (485) days for a COMP prosthesis. We report that U.S. Service members who were prescribed an ESR-AR or a COMP were more likely to experience a longer time between injury and amputation (> 90 days), and amputation to prescription (> 204 days). Therefore, it is evident that timing within the prescription procedure can significantly influence initial prescription patterns, however there are many factors that may influence the timing of such events. For example, many combat-related amputations occur acutely. However, previous work has documented that out of 348 major limb amputations, 15% occurred three months post-injury (12). Our outcomes also align with those of Krueger and colleagues, who demonstrated that 10% of U.S Service members underwent an amputation greater than 90 days after the date of injury (10). Previous literature has also revealed that amputations three months post-injury were associated with poor functional outcomes two years post-amputation (16). Identifying an optimal ankle-foot prosthesis at initial prescription is crucial to the rehabilitation process post-amputation; it has been associated with increased physical functioning, vitality and satisfaction, and reduced bodily pain (18). Nevertheless, prosthetic prescription has been identified as one of the major issues individuals encounter during the rehabilitation process (28). Our findings demonstrate that the interval between injury and prescription and amputation and prescription may influence initial ankle-foot prescription patterns in U.S. Service members. Of note, although not a primary aim included within our analysis, it is plausible that a Service member's specific rank/designation (e.g., special ops) may also influence the timing between prosthesis prescription (and other care) relative to injury and/or amputation. The decision-making process on whether to amputate or attempt limb salvage is multi-faceted and complex, considering numerous factors, such as the patient's pre-injury status, injury factors, and available resources (13, 29). The timing between initial prosthesis prescription and relative to injury/or amputation may be impacted by the physical and psychological status of the individual. Although not part of the aim of this investigation, secondary health conditions and complications following lower limb amputations are common (30). Therefore, it is plausible that these factors may reflect the wide range of timing variables in our sample. Considering individual factors such as the timing of injury and amputation relative to initial prosthesis prescription may enhance initial ankle-foot prosthesis prescription by attenuating potential prosthesis-related issues U.S. Service members may encounter during the rehabilitation process. The amputation etiology also significantly influenced the initial prosthesis prescription in U.S. Service members. Combat blast injury was the most reported injury resulting in amputation within the current sample. This aligns with previous literature reporting blast injury as the common cause of amputation within this population sector (2). For example, Farrokhi and colleagues report that during a 17-year surveillance period in a U.S. Service member population, 90.6% of amputations were caused by blast injury (2). Here, 84% of U.S. Service members experiencing a combat-blast injury were initially prescribed an ESR-NA prosthesis, 12% were prescribed an ESR-AR prosthesis, and 4% were prescribed a COMP prosthesis. There was a greater than 99% probability that those injured by a combat blast would be prescribed an ESR-NA over an ESR-AR or over a COMP. There was also an 85% and >99% probability that those amputated as a result of dysvascular disease and cancer, respectively, would be prescribed an ESR-AR over an ESR-NA. While individuals with lower limb loss exhibit marked heterogeneity concerning the etiology of amputation, even within a U.S. Service member population, the cause of amputation may vary. It is also crucial to consider the range of duty status (active, reserve, dependent, and retired) within this sample (Table 1) and how this may relate to amputation etiology. Dysvascular disease has been reported to be the leading cause of amputation in the civilian population, particularly with advancing age (1). The mechanistic underpinnings of the injury are crucial to understanding aspects of the prosthetic rehabilitation process, especially as they influence the particular type of prosthesis an individual may receive. Our model, incorporating the time from injury to initial prosthesis prescription, time from amputation to initial prosthesis prescription, sex, and amputation etiology as predictors, correctly predicted and classified 72% of all initial prosthesis types prescribed to U.S. Service members. Within each prosthesis type, our model correctly classified 71% of ESR-NA prostheses, 71% of ESR-AR prostheses, and 38% of the COMP prosthesis. The lower success rate in classifying the COMP prosthesis may result from the smaller number of U.S. Service members in our sample that were prescribed a COMP prosthesis (*N* = 8) compared to other prosthesis types. This is perhaps unsurprising, as ESR devices are the current gold standard for prescription within the Department of Defense (DoD) (20). ESR devices can offer several advantages, such as increasing walking speed, reducing the energetic cost of walking, improving elastic response, and aiding propulsion (21–23). Furthermore, it is also important to highlight that limited commercial options are available when considering powered propulsion COMP devices. However, it is encouraging that four patient-specific characteristics could correctly predict initial prosthesis type. These findings indicate that individual characteristics may relate to specific ankle-foot prosthesis prescription patterns among U.S. Service members. Our study also revealed that initial ankle-foot prosthesis prescription patterns may be impacted by sex. Females exhibited an increased probability and were more likely to be prescribed a COMP device than an ESR-NA device. Although generally consistent with the larger Service member and veteran populations, females comprised just 6.3% of our total sample (*N* = 11/174). Considering ankle foot-foot prosthesis category, 25% of COMP were females, 10.7% of ESR-AR were females, and 4.3% of ESR-NA were females. While the model does depict an increased likelihood of females receiving a COMP prosthesis, it is imperative to interpret these findings with caution due to both the small number of females and overall prescriptions of a COMP prosthesis (*N* = 8) compared to other prosthesis types. Nonetheless, females with limb loss present with unique considerations for prosthesis prescription and additional work is certainly needed to improve outcomes for this population. While the main focus was toward describing initial ankle-foot prosthesis prescription patterns in active-duty U.S. Service members, we ultimately identified several patients outside of this designation (e.g., dependent, retired) who received care during the study window. Duty status at injury or amputation was not different at initial device prescription (Table 1 highlights how ankle-foot prosthesis prescription may be influenced by duty status). When examining the distribution across prosthetic foot type for the subset who were active-duty U.S. Service Members (*n* = 155), as in the full sample, time from injury to prescription, time from amputation to prescription, and amputation etiology predicted first device prescribed. However, in this subset of active-duty Service members, time from injury to amputation also predicted first device prescribed, and sex did not. Of note, the primary amputation etiology for non-active-duty personnel was dysvascular etiology compared to combat blast injury for active duty (Table 1). While the scope of this paper was to provide a necessary first step in characterizing ankle-foot prosthesis prescription patterns primarily in active duty U.S. Service members, future studies should expand to other patient groups and clinical settings, particularly for improving generalizability to the veteran and civilian sectors. Furthermore, while the focus of our study was solely on initial device prescription, most of the Service members within our study went on to receive additional prescriptions. While outside the scope of this particular study, future work should examine how ankle-foot device prescription altered for each individual over time as this could help guide studies using outcome measures or end user feedback. Several other key considerations concerning ankle-foot component selection were not included in our study but are important to note. First, it is crucial to consider the functional status and mobility needs of individuals following limb loss. For example, many of the Service members in our study may be higher functioning and seeking to return to service as soon as possible. Therefore, it is plausible that they may require an ankle-foot device with particular features that allows them to negotiate different environments. Second, the prescription process is incredibly complex; individuals can often present with comorbid health conditions and experience secondary complications alongside prosthesis fitting. Therefore, it is critical to interpret our results cautiously as we did not report on such factors vital within the prescription procedure. It is plausible that a longer time between injury and prescription and amputation and prescription may reflect the physical and psychological state of the Service members. Third, we report only on the initial ankle-foot device prescribed to Service members, which may not reflect optimal (or only) device selection.

### 4.1. Study limitations

There are several limitations to consider with our study. First, without subsequent device use and clinical outcomes we are unable to deduce if specific prescriptions were optimal (improved satisfaction, quality of life, and physical function), nor if other clinical or institutional factors may have driven these initial prescription patterns. Future work should explore how such relationships may influence aspects of physical function, mobility, and quality of life. However, identifying shared characteristics in people prescribed specific ankle-foot devices may aid in better understanding of the role of these characteristics in clinical decision-making and in guiding future research initiatives. Further, due to the retrospective nature of the study design, incomplete or missing data was unable to be recovered, leading to the exclusion of many participants (118 participants were excluded due to missing initial prosthesis type, initial prosthesis prescription date, injury date, or amputation date). Additionally, collinearity between amputation etiology (combat blast) and age at device prescription likely confounded the odds ratios and confidence intervals for the combat blast in the logistic regression. Our analysis was restricted to individuals who received post-amputation care at Walter Reed National Military Medical Center; therefore, these relationships may not necessarily be generalized across the Department of Defense, Veterans Affairs, or to civilian practice. U.S. Service members also typically receive extensive therapies, training, and rehabilitation, both pre- and post-prosthesis, and including device- and activity- specific training. Although not the focus of this study, these are critical factors when considering optimal use/success of a prosthesis, and likely a major difference between population/care sectors. Prospective studies examining prothesis prescription patterns should utilize a more comprehensive approach, in particular obtaining both functional and patient-reported outcomes. While our study did not compare the association between the Medicare Functional Classification Levels (i.e., K-Level) and initial ankle-foot device prescription, such a functional index is not explicitly required within the DoD setting, contrary to the private sector. This study may also be limited, considering we included three categories of ankle-foot types. Future work should seek to explore the relationships between initial prescription patterns and a more specific/precise categorization of ankle-foot types.

## 5. Conclusions

This study identified patient-specific characteristics such as sex, time between injury and amputation, and amputation etiology influencing (first) ankle-foot prosthesis prescription in U.S. Service members and dependents receiving care within the Department of Defense at Walter Reed National Military Medical Center. Our findings suggest that younger Service members are more likely to receive an initial ESR-NA prosthesis, a shorter time between injury and amputation and a non-combat blast injury increases the odds of initially receiving an ESR-AR prosthesis. While more work is needed to track subsequent prosthesis use and outcomes, our study reinforces the importance of considering patient-specific factors during prosthesis prescription, to ensure an optimal first device prescription and post-amputation quality of life.

## Data Availability

The raw data supporting the conclusions of this article will be made available by the authors, without undue reservation.
